# Antimicrobial activity of Nd: YAG irradiation, laser activated photodynamic therapy and passive ultrasonic irrigation on *Enterococcus faecalis* biofilms (an ex vivo study)

**DOI:** 10.1186/s12903-026-08651-6

**Published:** 2026-05-26

**Authors:** Salma M. El Gazar, Rania N. El Backly, Walid A. Lotfy, Sybel M. Moussa, Ahmed M. Mobarak

**Affiliations:** 1 Conservative Dentistry Department, Faculty of Dentistry, Alexandria University, Alexandria, Egypt; 2 Tissue Engineering Laboratories, Faculty of Dentistry, Alexandria University, Alexandria, Egypt; 3 Department of Microbiology, Faculty of Dentistry, Pharos University, Alexandria, Egypt

**Keywords:** Root canal disinfection, Irrigation, aPDT, Nd: YAG laser irrigation, Passive ultrasonic irrigation, Sodium hypochlorite

## Abstract

**Aim:**

To evaluate the antimicrobial effects of Nd: YAG laser irradiation, antimicrobial photodynamic therapy (aPDT), and passive ultrasonic irrigation (PUI) in comparison with conventional irrigation with Sodium hypochlorite (NaOCl) in root canals infected with *Enterococcus faecalis *biofilms.

**Materials and methods:**

Sixty-nine single rooted mandibular premolars were accessed and instrumented till master apical file #40/04. After sterilization of the teeth, the root canals were infected with E. faecalis for 3 weeks. The teeth were randomly divided into five groups according to the method of disinfection (n=13 each): group I: irrigation with distilled water; group II: irrigation with 2.5% sodium hypochlorite (NaOCl) with side vented needle; group III: PUI activation for 2.5% NaOCl; group IV: Nd:YAG laser activation for 2.5% NaOCl , group V: aPDT with 0.01% methylene blue as the photosensitizer and laser irradiation (λ635nm, power 400mW, 5min). The four remaining teeth were used as control groups (n=2 each): negative control (without bacteria) and positive control (without treatment). Colony forming units (CFU) were then counted and Scanning Electron Microscope (SEM) analysis was performed to evaluate *Enterococcus faecalis* biofilm reduction. Results were analyzed using the Kruskal–Wallis test followed by Bonferroni‑adjusted pairwise comparisons.

**Results:**

The bacterial counts in the NaOCl needle irrigation, PUI, and Nd: YAG laser groups were significantly lower than those of the untreated positive control and distilled water groups. Furthermore, they were also significantly lower than those of the aPDT group which did not show any significant reduction in bacterial count compared to the positive control nor the distilled water groups.

**Conclusion:**

Adjunctive irrigation using Nd:YAG laser and PUI increased bacterial reduction, however, they did not significantly enhance the antibacterial effects of NaOCl when used alone in this specific tooth model. Furthermore, antimicrobial photodynamic therapy with methylene blue failed to show any noticeable antimicrobial effects.

**Supplementary Information:**

The online version contains supplementary material available at https://doi.org/10.1186/s12903-026-08651-6.

## Significance

The findings of this study indicate that NaOCl, NaOCl + PUI and NaOCl + Nd: YAG disinfection techniques achieved near-complete microbial elimination, whereas aPDT was largely ineffective under the tested conditions, performing comparably to distilled water.

## Background

Root canal instrumentation is primarily viewed as a means of providing irrigants access to the apical root canal system, allowing them to perform most of the cleaning and disinfection [1]. Mechanical instrumentation is seldom capable of touching all the root canal walls particularly in cases where there is complex root canal anatomy such as the presence of lateral and accessory canals, isthmuses, fins, c-shaped canals and even wide oval canals. The latter may represent unique challenges as the percentage of untouched walls may indeed be larger than in narrower canals adding more emphasis on the crucial role of effective root canal irrigation [2]. Indeed, while the coronal and middle thirds of root canals maybe easily accessible to endodontic disinfection strategies, reaching the intricate apical third anatomy poses further challenges.

Therefore, adjunctive methods of irrigant activation have been recommended to improve and enhance the effect of irrigants to overcome such limitations. Such methods are inherently critical in cases with persistent apical periodontitis particularly related to the presence of resistant microbial species such as *Enterococcus faecalis* (E.faecalis). E. faecalis is a facultative anaerobic bacteria that is extremely resistant to calcium hydroxide and traditional chemo-mechanical preparation [3–5]. These traits appear to be linked to its ability to enter dentinal tubules deeply and withstand harsh environmental conditions. E. faecalis is the most frequent bacteria found in teeth which need retreatment because of failed primary root canal treatment [6]. Since preventing or treating apical periodontitis is the main objective of root canal therapy, all bacteria—both planktonic and biofilm—should be eliminated from the root canal system. Microorganisms are successfully protected by biofilms; hence the disintegration of the biofilm matrix structure is therefore necessary to provide effective disinfection [7].

The use of ultrasonic activation of NaOCl in endodontic procedures has been shown to allow the irrigant to penetrate into lateral canals, dentinal tubules, and un-instrumented areas in wide canals, significantly reducing bacterial populations and removing hard tissue debris from the root canal [8]. By creating cavitations and acoustic microstreaming in the intracanal irrigant, passive ultrasonic irrigation (PUI) may aid in the elimination of endodontic biofilms and the removal of intracanal smear layer [9].

Additionally, laser systems have been recommended in endodontics to improve the agitation of the irrigant and, consequently, intracanal disinfection [6]. Microorganisms can be eliminated by laser light because they can reach inaccessible areas. While Er: YAG lasers are frequently utilized for surface ablation due to high water absorption, their effective bactericidal depth is limited to 100–200 μm. Numerous investigations in contrast, have demonstrated that the Nd: YAG laser may penetrate deeper into the dentin, reducing the invasion of bacteria [10, 11]. Nd: YAG’s antimicrobial impact is based on thermally heating the bacterial environment and local heating within the bacteria themselves (through chromophores that are laser-sensitive) [12].

The antimicrobial photodynamic therapy (aPDT), also known as photoactivated disinfection (PAD), may also be achieved using laser irradiation. It uses a low-level Diode laser (620–700 nm) and a non-toxic photosensitive dye like methylene blue [10]. Molecular oxygen is converted into a highly reactive oxygen or singlet oxygen (¹O₂) molecule as a result of the interaction between the photosensitizer and the laser light, which damages the bacterial membrane and DNA. The photosensitizers are chosen so that they have a particular affinity for the bacterial membranes without impacting the viability of the host cells [12].

Therefore, the research question of the current study was whether the use of Nd: YAG irradiation, laser activated aPDT and passive ultrasonic activation would effectively reduce *Enterococcus faecalis* biofilms in an ex vivo infected root canal model. The null hypothesis of this study was that there would be no difference in the antimicrobial effects between Nd: YAG irradiation, aPDT and passive ultrasonic activation when compared to conventional needle irrigation in single root canals infected with *Enterococcus faecalis*.

## Materials and methods

### Study design

**Fig. 1:**
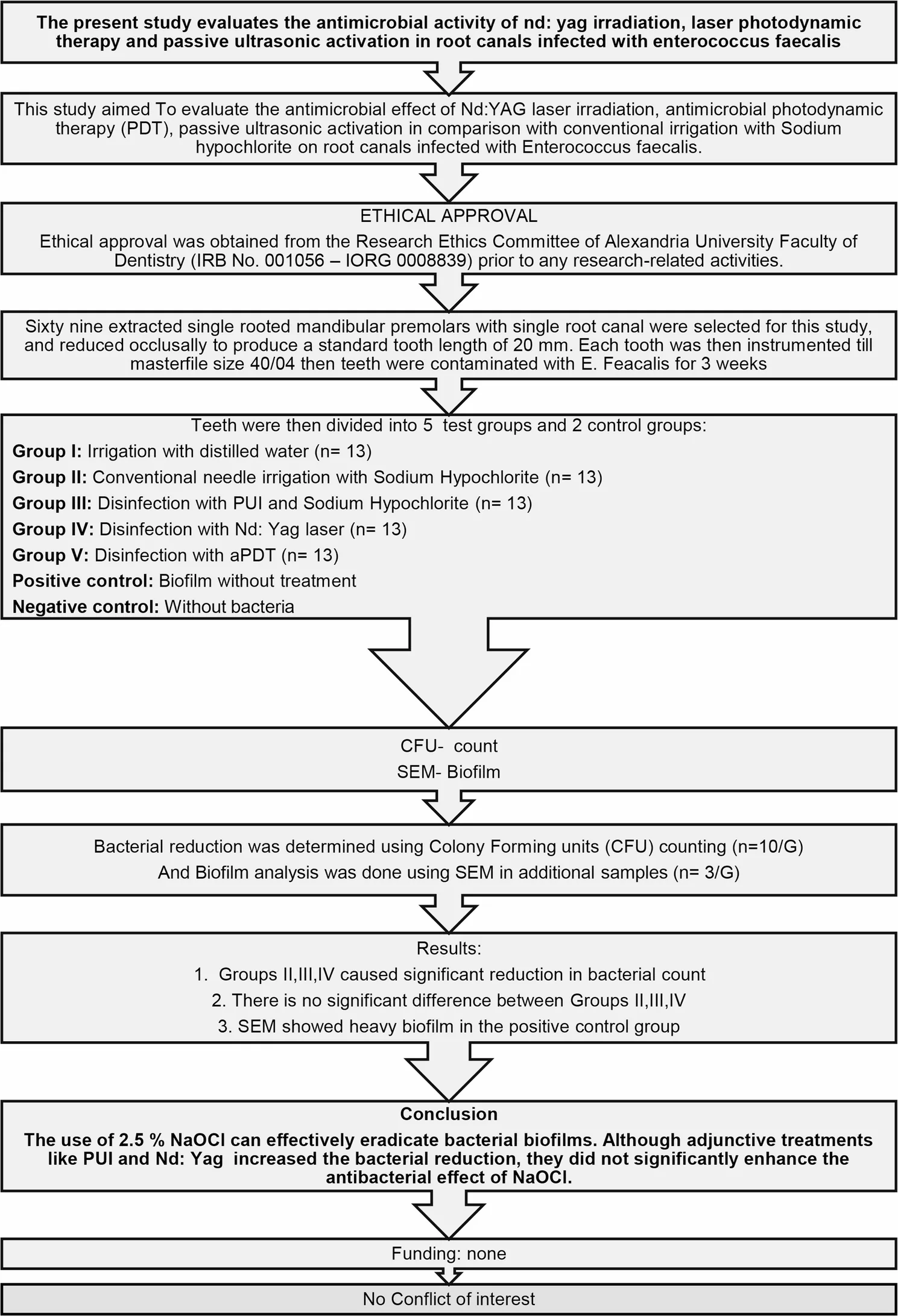
Flowchart of the study design, methodology, grouping, and results in the current study according to PRILE guidelines

The current laboratory in vitro study was conducted and reported in full accordance with the Preferred Reporting Items for Laboratory studies in Endodontology (PRILE) 2021 guidelines Fig. 1 [13].

This study was conducted on 69 extracted mandibular premolar teeth collected from the out-patient clinic of the Oral and Maxillofacial Surgery Department, Faculty of Dentistry, Alexandria University. The teeth were extracted from adults aged 18–50 years due to periodontal disease or orthodontic treatment.

Following the anonymization of these samples, the Research Ethics Committee approved their use for research purposes. The research protocol was approved by the Research Ethics Committee of Alexandria University, Faculty of Dentistry (IRB No. 00010556 ; IORG 0008839; Approval no. 0818 − 12/2023).

### Sample size

Sample size was calculated using G*Power (Version 3.1.9.7) assuming 80% study power and a 5% alpha error based on previously published data. Bago Jurič et al. [14] reported mean (SD) *Enterococcus faecalis* colony forming units (CFU) of 5.87 (1.94) × 10⁴ for photodynamic therapy and 20 (5.75) × 10⁴ for Nd: YAG laser irradiation. In addition, Toljan et al. [15] reported mean (SD) CFU values of 8.98 (7.44) × 10¹ following passive ultrasonic activation and 10.5 (5.39) × 10¹ for the positive control group. Based on comparison of means and using the highest reported standard deviation to ensure statistical power, the minimum required sample size was calculated to be 9 samples per group, which was increased to 10 to account for potential laboratory processing errors. Accordingly, for five experimental groups, the total required sample size was 50 samples [16].

Additional specimens were included to allow for scanning electron microscopy (SEM) evaluation. As SEM analysis was qualitative in nature, 3 additional samples per group were allocated for SEM and were not included in the statistical analysis. Furthermore, 2 positive and 2 negative control samples were included for validation of antimicrobial efficacy.

Therefore, the total number of specimens used in the study was 69. Statistical analysis was performed exclusively on the calculated sample size (*n* = 50), ensuring adequate study power, while the additional samples were used solely for qualitative and confirmatory assessment.

### Sample selection

Freshly extracted single-rooted mature mandibular sound premolars which were previously stored in saline (to ensure there is no antibacterial effect) and having roots with Vertucci Type I canal configuration and with medium root curvature, were included [6, 17, 18].

The excluded teeth were the ones which were previously stored in formalin solution, endodontically treated teeth and teeth with multiple root canals, root caries, resorption defects, fractures, or cracks [19].

### Root canal preparation

Access cavity preparation was performed using rose head and safe end burs (Dentsply Sirona, Ballaigues, Switzerland). A #10 K-File (Dentsply Maillefer, Ballaigues, Switzerland) was inserted into the root canal until it was seen at the apical foramen and retracted to be flushed with the apex. One millimeter was subtracted from this length and was taken as the working length (WL). The teeth were reduced occlusally to produce a standard tooth length of 20 mm. All teeth were instrumented to the WL using stainless steel K-Files to size #20 followed by rotary files (Fanta Dental, Shanghai, China) up to size 40/4% taper. At every instrument change, the root canal was irrigated with 2 mL of 2.5% NaOCl and Glyde (Oxoid ltd, Basingstoke, Hampshire, England) was used as a lubricant [20]. As a final rinse 17% EDTA solution was delivered into the canal using a side vented needle 2 mm shorter than the working length, to remove the smear layer. Following the disinfection protocols, the canals were irrigated with 5 mL of 5% sodium thiosulfate (Piochem, Egypt) for 60 s to neutralize any carryover antimicrobial effect of the irrigant which may result in false negative results. The outer surfaces of the roots were covered with two layers of nail varnish to avoid external microbial contamination [21]. Each specimen was placed in a cryovial containing 500 µL of brain heart infusion (BHI) (Oxoid ltd, Basingstoke, Hampshire, England) broth, as a verification of sterility, specimens were subsequently autoclaved at 121 °C for 30 min [22].

**Fig. 2:**
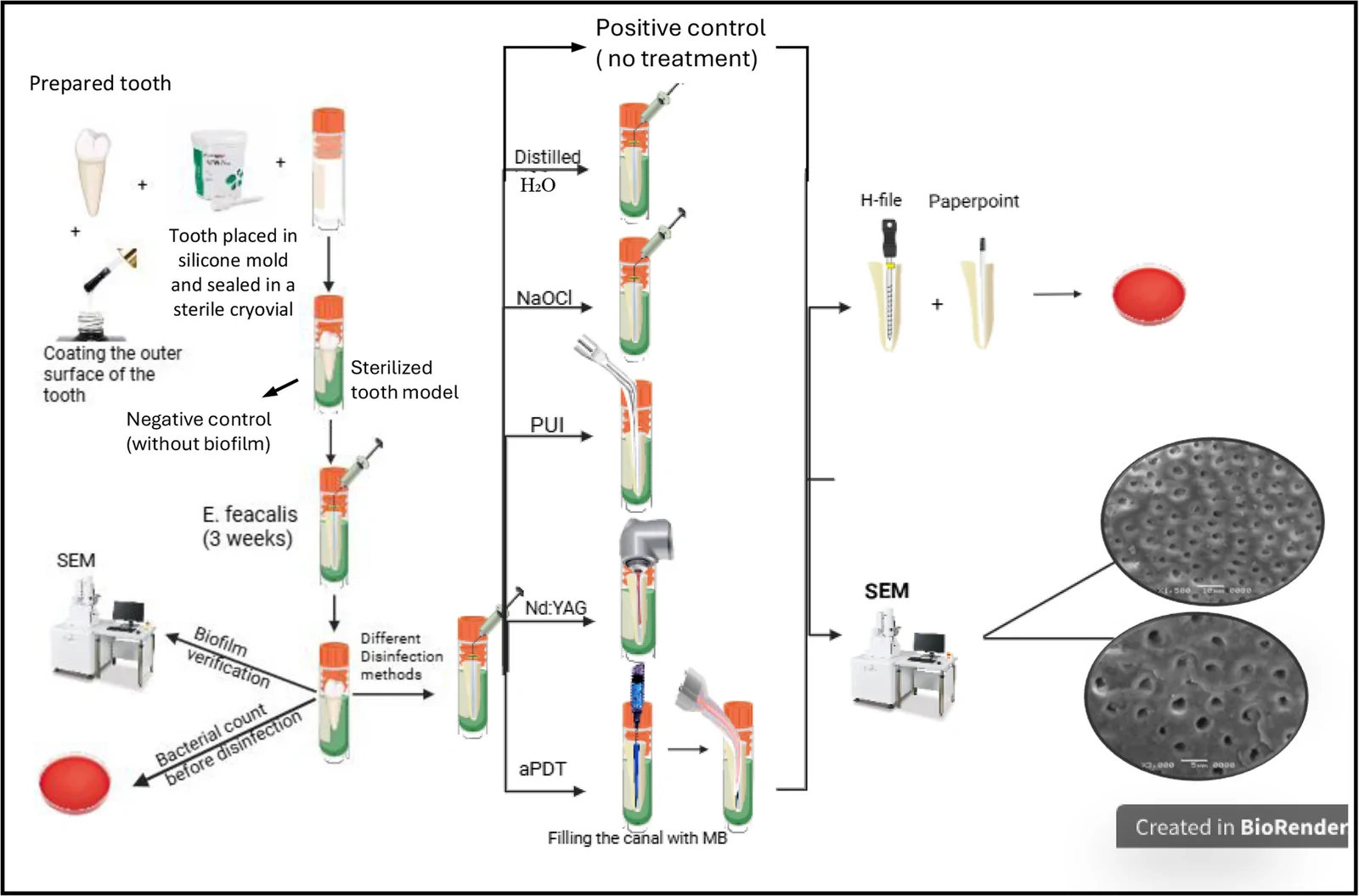
Schematic diagram representing the methodology executed in the study. Teeth were prepared, mounted in silicone molds then inoculated with *Enterococcus faecalis* to create mature biofilms. Samples were then randomly allocated to different irrigation protocols then CFUs were counted and residual biofilms examined using scanning electron microscopy

A customized model (Fig. 2) was assembled for each tooth to facilitate subsequent bacterial inoculation and sampling. Silicone impression material (Zetaflow condensation silicone, Zhermack, Badia Polesine, Rovigo, Italy) was expressed into a sterile cryovial and the teeth were mounted vertically in it. After silicone material setting, the interface with each tooth surface was sealed with cyanoacrylate resin. Then, the specimens were autoclaved for a second cycle to ensure sterility of the whole assembly [21, 23].

E. faecalis culture (ATCC 29212) (American Type Culture Collection, Manassas, Virginia, USA.) was grown on blood agar (Oxoid ltd, Basingstoke, Hampshire, England) plates at 37 °C for 24 h. Blood agar was used for the colony forming unit counting of E. faecalis instead of BHI since it is an enriched medium that provides essential growth factors and hemin, which can aid in the recovery of stressed E. faecalis cells more effectively than standard BHI agar [24]. Pure colonies of E. faecalis were suspended in 5 mL of sterile BHI broth. Each root canal was filled with the E. faecalis suspension. The inoculum inside each canal was replaced with fresh bacterial suspension every other day. All specimens were incubated for three weeks under aerobic conditions at 37 °C [25, 26]. To confirm the purity of the E. faecalis, random sampling was done for gram staining every week and examined using a light microscope [25]. An antibiotic sensitivity test was done after 3 weeks to ensure purity of the E. Faecalis.

After the incubation period, the number of the colony forming units (CFUs) was counted on the plates using a bacterial colony counter and 3 additional samples were taken for SEM examination to confirm bacterial biofilm formation [27, 28]. A total of 65 teeth were randomly divided into 5 test groups I-V (*n* = 13/group) according to the type of disinfection method used. The remaining 4 teeth were added: 2 negative controls (without bacteria) and 2 positive controls (biofilm without disinfection) for CFU counting and SEM examination.

### Different disinfection techniques

Sixty-five teeth were randomly divided according to the method of disinfection used. 

Group I: Irrigation with distilled water; Group II: Conventional needle irrigation with Sodium Hypochlorite; Group III: Disinfection with PUI and Sodium Hypochlorite; Group IV: Disinfection with Nd: Yag laser + NaOCl; Group V: Disinfection with aPDT and methylene blue (MB).

#### Group I : irrigation with distilled water (*n* = 13)

The root canals were irrigated with 5 ml distilled water for 60 s using a 30 gauge side vented needle (Becton Dickinson, Madrid, Spain) 2 mm shorter than the WL.

#### Group II: disinfection with NaOCl ( conventional needle irrigation) (*n* = 13)

The root canals were irrigated with 5 mL 2.5% NaOCl for 60 s using a 5 mL syringe and a 30 gauge side vented needle, placed 2 mm short of the WL [29, 30].

#### Group III: disinfection with PUI+ NaOCl (*n* = 13)

The canals were irrigated as mentioned in Group II then the PUI was performed using an ultrasonic activator (Ultra Mint Pro, Eighteeth) with the Ultrasonic tip E96 #20/02 (Guilin Woodpecker Medical instrument Co., Ltd.). The ultrasonic tip #20 was placed 1 mm short of the WL and kept centered in the canal then 2–3 mm apical-coronal movements were done for 20 s. The instrument was inserted passively and activated with a medium power of 40%-50% [31]. The passive irrigation technique with an intermittent flush consisted of applying 3 cycles of ultrasonic activation of the irrigant for 20 s each, so that each canal would be subjected to a total of 1 min of passive ultrasonic irrigation [29, 30, 32, 33].

#### Group IV: disinfection with Nd: YAG + NaOCl (*n* = 13)

The canals were irrigated as mentioned in Group II then the length of the root canals was transferred accurately to the flexible 300-µm optical fiber to ensure it reached the predetermined WL minus one millimeter. Only then, the laser (Nd: YAG laser1064 nm, 1.5 W, 15 HZ; AT Fidelis, Fotona, Slovenia) was activated, and the fiber non-initiated tip was guided from the apical area to the coronal area with rotary movements (spiral movements) and in contact with the root canal wall. The procedure was repeated three times for 20 s each with replenishment of NaOCl in between [10, 34]. The energy fluence delivered per canal was calculated as 30 J repeated three times, ensuring standardization among samples.

#### Group V: disinfection with aPDT (*n* = 13)

The photosentizing agent methylene blue 0.01% (Alpha Chemika - Methylene Blue (Sr. No. AL3055), India) was delivered inside the canal and left for 60 s as preradiation time, for bacterial staining and then irradiated continuously for 5 min [35]. This was performed using a Diode laser (Lasotronix, wavelength 635 nm, 400mW) with the fiber optic probe advanced to be 1 mm shorter than the WL with a 200 μm diameter fiber optic probe [6, 17]. The energy fluence delivered per canal was calculated as 120 J, ensuring standardization among samples.

### Quantification of bacterial colonies after disinfection with each method

At the end of each treatment period, for all root canals a sterile Hedstrom file #40 was used to scrape the dentinal walls for 20 s. Then the canals were filled with 1 ml sterile saline solution, and the root canal content was aspirated with a sterile insulin syringe and transferred to an Eppendorf tube. Then, three sterile paper points size #40 were used to collect the remaining fluid and the dentin chips in the root canals. Each paper point was left inside the canal for 1 min. The contaminated paper points were then placed into a microcentrifuge tube containing 1 mL BHI [14, 25]. BHI broth was selected instead of PBS as the collection and transport medium to avoid loss of viability of fastidious or damaged bacterial cells prior to culturing, potentially resulting in false-negative culture results [28].

The saline which was previously aspirated from the canals was added to the tube with BHI. The tubes were vortexed for 1 min. Subsequently, 1 µL from the suspension was smeared on a blood agar plate using a sterile calibrated plastic loop and then the plate was incubated at 37 °C for 48 h [24]. After the incubation period, the number of colony forming units (CFUs) was counted on plates using a bacterial colony counter and the count of CFU/mL for each plate was calculated [28].

### SEM examination

Three random samples (other than the samples used for bacterial quantification) were selected from each group for SEM. The teeth were sectioned longitudinally into 2 halves, which were examined using SEM for biofilm descriptive evaluation [6]. Longitudinal grooves were established on the buccal and lingual surfaces via a diamond disc, maintaining sufficient dentinal thickness to avoid canal penetration. Subsequently, the roots were separated into two segments through the application of a chisel and hammer [36].

Each half of the specimen was soaked in a fixative solution (4:1 ratio) of 2% glutraldehyde, 2.5% formaldehyde at pH 7.5 for 24 h then washed with PBS solution 4 times 20 min each, then dehydrated using gradient concentrations of ethanol (30% to 90% ) for 5 min each then dehydrated in 100% ethanol 2 times for 10 min each. Specimens were dried, mounted onto aluminum stubs using carbon tape, and sputter coated with gold layer 20 nm and visualized with a SEM. (JEOL JSM-5510LV Scanning Electron Microscope; Jeol Ltd., Tokyo, Japan) [37].

Representative images were acquired after thoroughly scanning the sample from the coronal till the apical third with a minimum of 3–5 images per sample for scoring assessment [38].

For quantification purposes, a modification of the four-score scale used by Ordinola-Zapata et al. was implemented as follows [39, 40] :

- Score 1: Clean dentin or residual isolated microbial cells that cover <0-25% of the dentin. Absence of residual bioﬁlm layers.
- Score 2: Residual isolated microbial cells cover 25-50% of the dentin. There is absence of residual bioﬁlm layers.
- Score 3: Bioﬁlm structures and microbial cells can be identiﬁed covering 50-75% of the dentin.
- Score 4: Bioﬁlm structures and microbial cells can be identiﬁed covering 75–100% of the dentin.

The SEM images taken from the samples and the biofilm areas were traced using imageJ software (NIH, USA). After the scale was set and the biolfilm surface area was traced, all surface areas were added, and the final result was then divided by the total surface area and multiplied by 100 to get the percent of biofilm covering the dentin surface.

### Statistical analysis

Statistical analysis was performed using IBM SPSS Statistics for Windows, version 26.0 (IBM Corp., Armonk, NY, USA). Normality was assessed using the Shapiro–Wilk test, which indicated non-normal distribution of the data (*p* < 0.05). Accordingly, non-parametric tests were applied. Results are presented as mean ± SD along with 95% confidence intervals (CI), with median and interquartile range (IQR). Comparisons between groups were performed using the Kruskal–Wallis test, followed by Bonferroni-adjusted post hoc pairwise comparisons to control for multiple testing. In addition to p-values, effect sizes were calculated to quantify the magnitude of differences between groups. For the Kruskal–Wallis test, eta-squared (η²) was estimated using the formula η² = (H − k + 1) / (n − k), where H is the Kruskal–Wallis statistic, k is the number of groups, and n is the total sample size. For pairwise comparisons, rank-biserial correlation (r) was used. Statistical significance was set at α = 0.05.

## Results

### Effects of different disinfection protocols on bacterial CFU count reduction

Based on CFU counts, all experimental groups demonstrated a statistically significant reduction in bacterial load compared with both the positive control and the distilled water groups (*p* < 0.05) (Table 1). The greatest reduction in bacterial counts was observed in the Nd: YAG laser and in the Passive Ultrasonic Irrigation (PUI) groups, followed by Sodium Hypochlorite (NaOCl) alone with no significant differences between these three groups. The Antimicrobial Photodynamic Therapy (aPDT) group exhibited significantly less bacterial reduction than the latter 3 groups. The amount of bacterial reduction observed in the aPDT group was not significantly better than either of the positive control nor distilled water groups. Regarding percent reduction (Table 1), the Nd: YAG laser, PUI, and NaOCl groups achieved substantial decreases in viable bacterial counts, with mean reductions exceeding 93%, confirming their high disinfection efficiency. The Nd: YAG laser achieved the greatest mean reduction (− 97.7 ± 5.0%), followed closely by PUI (− 95.8 ± 8.0%) and NaOCl (− 93.4 ± 10.0%). In contrast, aPDT (− 24.6 ± 21.4%) and distilled water (− 46.8 ± 27.4%) exhibited markedly lower reductions, with no significant difference between them.

Post-hoc analysis (Table 1) confirmed statistical similarity among NaOCl, PUI, and Nd: YAG, yet each significantly outperformed aPDT and the control group. When expressed as log₁₀ reductions, the same ranking was maintained, reinforcing the strong bactericidal efficacy of NaOCl, PUI, and Nd: YAG. Overall, these findings indicate that all three primary disinfection techniques achieved near-complete microbial elimination, whereas aPDT was largely ineffective under the tested conditions, performing comparably to distilled water. 

**Table 1:** Comparison of E. Faecalis CFU between the study groups

		Distilled water	NaOCl	PUI	Nd: Yag laser	aPDT	Positive Control	P value
Count	Mean ± SD	290.40 ± 159.98	31.64 ± 45.47	21.85 ± 44.27	10.50 ± 22.42	391.40 ± 121.24	525.00 ± 111.25	<0.001*
	Median (IQR)	286.00 (156.00, 413.13) a	3.00 (0.00, 77.00) b	0.00 (0.00, 25.38) b	2.00 (0.00, 8.75) b	394.00 (296.38, 452.75) a	533.50 (419.63, 621.88) a	
Log_10_	Mean ± SD	2.40 ± 0.26	0.79 ± 0.92	0.58 ± 0.83	0.49 ± 0.67	2.57 ± 0.13	2.71 ± 0.09	<0.001*
	Median (IQR)	2.45 (2.19, 2.62) a	0.48 (0.00, 1.89) b	0.00 (0.00, 1.21) b	0.29 (0.00, 0.92) b	2.60 (2.47, 2.66) a	2.72 (2.62, 2.79) a	

Kruskal Wallis test was used

The effect size for the overall comparison: η² = 0.80

*SD* Standard Deviation, *IQR* Interquartile range

a-b: different letters denote statistically significant differences between groups using Bonferroni correction

*statistically significant at p-value <0.05

**Table 2:** Comparison of percent reduction of CFU between the study groups

		**Distilled water**	**NaOCl**	**PUI**	**Nd:Yag laser**	**aPDT**	**P value**
Count	Mean ± SD	-46.76 ± 27.38	-93.40 ± 9.98	-95.83 ± 8.02	-97.66 ± 4.99	-24.56 ± 21.41	<0.001*
	Median (IQR)	-47.71 (-63.17, -25.09) a	-99.33 (-100.0, -83.31) b	-100.0 (-100.0, -93.97) b	-99.53 (-100.0, -97.95) b	-23.63 (-45.59, -6.11) a	
Log10	Mean ± SD	-12.32 ± 9.37	-70.65 ± 34.37	-78.77 ± 30.67	-81.34 ± 25.06	-5.12 ± 4.90	<0.001*
	Median (IQR)	-10.08 (-16.49, -5.06) a	-82.02 (-100.0, -27.86) b	-100.0 (-100.0, -53.99) b	-89.06 (-100.0, -65.69) b	-4.34 (-9.61, -1.04) a	

Kruskal Wallis test was used

The effect size for the overall comparison: η² = 0.84

*SD* Standard Deviation, *IQR* Interquartile range

a-b: different letters denote statistically significant differences between groups using Bonferroni correction

*statistically significant at p-value <0.05

**Table 3:** Post-hoc comparisons of percent reduction of CFU between the study groups

**Group**	**Compared to**	**Count**	**Log**_**10**_
		**P value**	
Distilled water	NaOCl	<0.001*	<0.001*
	PUI	<0.001*	<0.001*
	ND:Yag laser	<0.001*	<0.001*
	aPDT	1.00	1.00
NaOCl	PUI	1.00	1.00
	ND:Yag laser	1.00	1.00
	aPDT	<0.001*	<0.001*
PUI	ND:Yag laser	1.00	1.00
	aPDT	<0.001*	<0.001*
ND:Yag laser	aPDT	<0.001*	<0.001*

*Statistically significant using Bonferroni corrected significance level

### SEM of E. faecalis biofilms

**Fig. 3:**
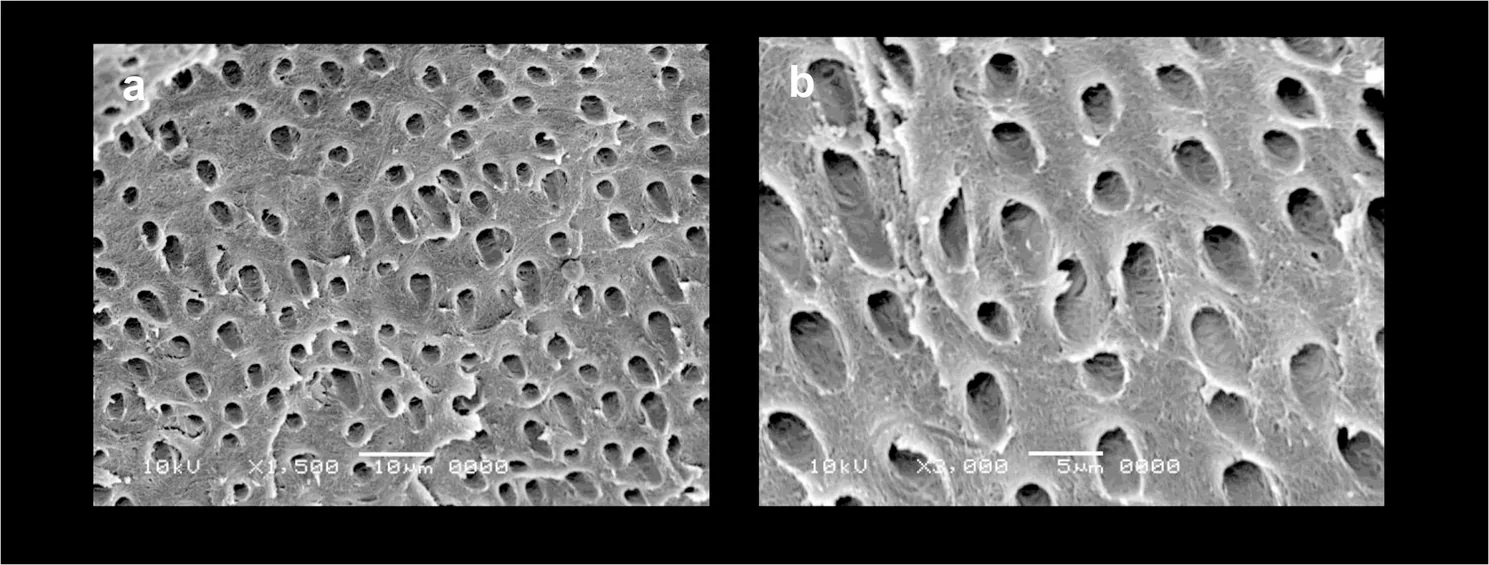
SEM images of a representative negative control sample showing clean dentine surface and open dentinal tubules; **a** at 1500x and **b** at 3000x magnification

SEM examination of the negative control group samples (Fig. 3a, b) showed a clean dentin surface and open dentinal tubules.

**Fig. 4:**
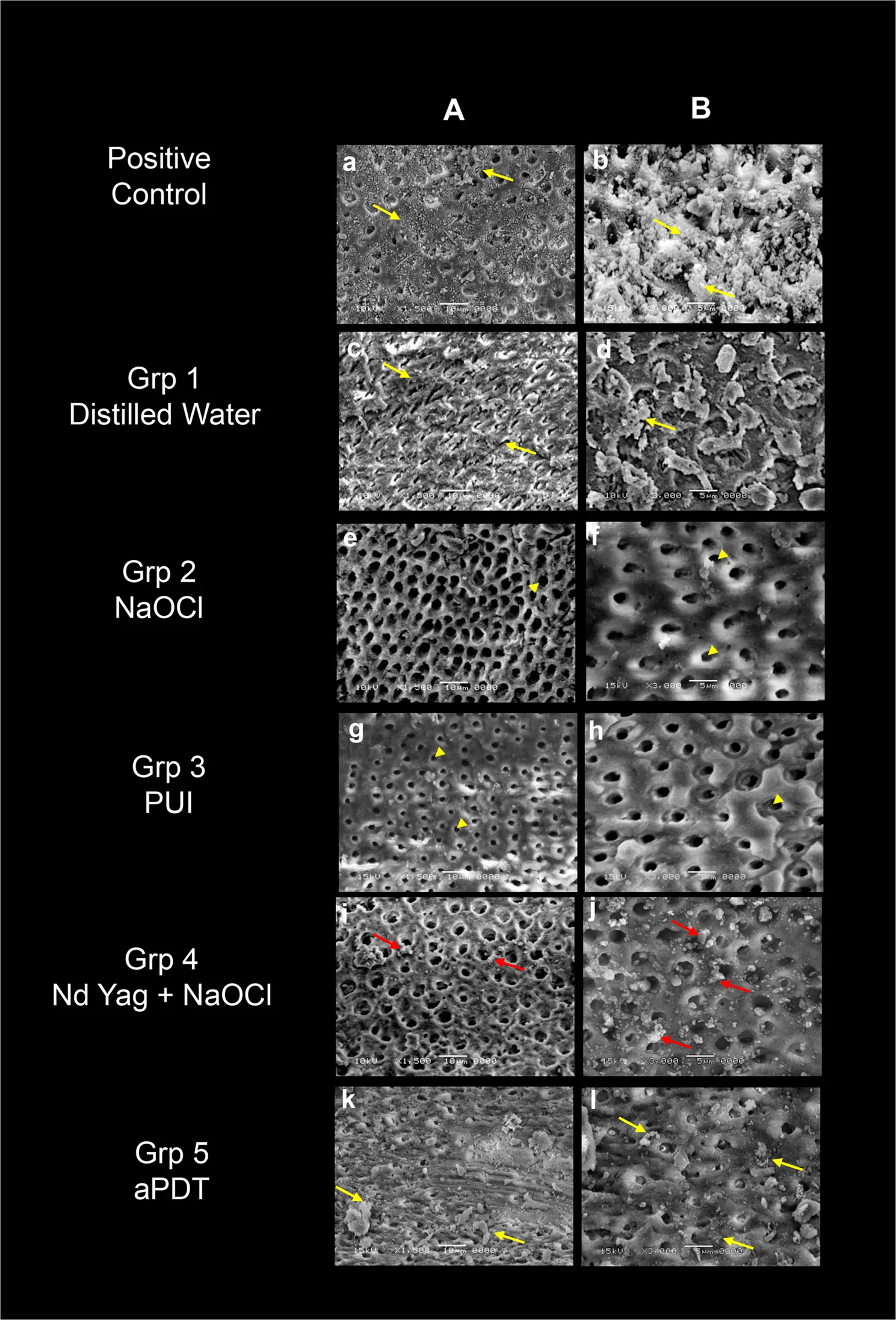
Representative SEM images of 3-week-old *Enterococcus faecalis* biofilms on the luminal surface of sectioned mandibular premolar teeth treated with different irrigation protocols. **a**, **b**, **c**, **d**, **k**, **l** showing mature, uniform biofilms covering most of the dentine surface of the positive control group in (**a**, **b**), the distilled water group (Grp1) in (**c**, **d**) and the aPDT group (Grp5) in (**k**, **l**); (**e**, **f**, **g**, **h**) showing little or no residual biofilm in the NaOCl group (Grp2) (**e**, **f**) and the PUI group (Grp3) (**g**, **h**); (**i**, **j**) showing some residual biofilm in the Nd: YAG group with some crystalline structures that could be due to a superficial melting and recrystallization effect on dentine (Grp4). Column A represents x1500 magnification for all groups whereas column B represent magnifications x3000. Yellow arrows point to dense bacterial biofilms, while yellow arrowheads point to open and clean dentinal tubules. Red arrows highlight the morphological changes and deposits on the dentinal surface caused by the Nd: YAG laser treatment

The Positive control samples (Fig. 4a, b) revealed a dense, mature, and uniformly distributed bacterial biofilm layer completely covering the dentinal surface. In the distilled water (Fig. 4c, d) and aPDT (Fig. 4k, l) groups, thick and cohesive biofilm structures were still evident with almost continuous surface coverage occluding the dentinal tubules.

The Nd: YAG + NaOCl group (Fig. 4i, j) displayed some residual bacterial biofilms, with scattered crystalline structures and debris. Additionally, large areas showing open and clearly visible dentinal tubules could be identified. Specimens treated with NaOCl alone (Fig. 4e, f) and NaOCl combined with PUI (Fig. 4g, h) exhibited the highest degree of biofilm disruption and surface cleanliness. These groups were characterized by minimal debris, with the majority of the surface revealing patent dentinal tubules showing sharp circular appearance which is also an indication of cleanliness. While the NaOCl + Nd: YAG group (Fig. 4i, j) demonstrated effective biofilm reduction and open dentinal tubules, the dentinal surface was obscured by widespread crystalline precipitates.

According to this modified scoring system used and applied on 3 samples from each group the scores were as follows: Positive control group, Distilled water group and aPDT received a Score 4. In the NaOCl group, 2 out of 3 samples got score 1 and the third sample got score 2. The PUI group and the Nd: YAG group received Score 1 in all 3 samples.

## Discussion

The current study evaluated the efficacy of PUI, Nd: YAG laser and aPDT compared to NaOCl conventional needle irrigation to eliminate or substantially reduce E. faecalis biofilms in an ex vivo model of infected single rooted mandibular premolar teeth. Although a recent systematic review highlighted the encouraging results of Nd: YAG laser activated irrigation as an adjunctive root canal disinfection technique, the lack of standardized parameters has often led to disparaging results and the difficulty in generalizing the outcomes of these studies [41]. The Nd: YAG wavelength (1064 nm) exhibits low absorption in water and hydroxyapatite, allowing for an optical penetration depth of up to 1,000 μm. In contrast, the Er: YAG wavelength (2940 nm) is highly absorbed by water, limiting its direct bactericidal effect to a superficial layer (approximately 100–200 μm). For this study, the objective was to target pathogens like E. faecalis that reside deep within the dentinal matrix beyond the reach of standard irrigation. Therefore Nd: YAG was the optimal choice for this study [11]. Furthermore, aPDT has also been suggested as an effective antimicrobial disinfection method in endodontics yet again it has been faced with inconsistent results when examined in preclinical and clinical studies. Therefore, the present study was designed to compare the antimicrobial effects of irrigation techniques based on the use of lasers namely, aPDT and Nd: YAG laser under standardized testing parameters and compare their effects to passive ultrasonic irrigation.

Mandibular premolars, with single root canals, were selected as the biofilm model in the present study as they often have oval shaped canals. These canals are challenging to be mechanically cleaned, because they can retain up to 35% of untouched surfaces [42]. The samples were standardized in length and preparation size to reduce potential bias in the study, and the specimens were inoculated with E. faecalis for three weeks to ensure the development of a mature biofilm as previously mentioned in many studies [20, 25, 43]. E. faecalis was selected for this study as it resists conventional treatments and poses a significant clinical challenge in eradicating root canal infections. Hence, it is widely regarded as a primary target for therapeutic strategies in majority of in vitro microbiological studies in endodontics [5]. While previous literature has utilized a range of 3–4 weeks, recent data from Hamed S.A. et al. [26] suggests that the biofilm is already physiologically mature at the three-week mark. We therefore chose this duration to capture the biofilm at its peak maturity.

In the present study, bacterial colony counts and biofilm evaluation using SEM were performed before and after the disinfection procedures. Colony-forming unit (CFU) enumeration remains a widely accepted and standardized technique for comparing the antibacterial efficacy of different endodontic disinfection protocols. Previous studies have shown a strong correlation between CFU recovery and both sampling methods using paper point and H-file techniques [14, 25]. The number of cells extracted from the canal can reliably reflect the quantity of bacteria adhering to dentin, and vice versa. These sampling approaches are considered adequate for detecting planktonic and superficially adherent microorganisms within the root canal lumen and on its walls hence they were utilized in the present study. Furthermore, complementing microbiological analysis, SEM provided direct visualization of the dentinal surface and allowed assessment of both biofilm morphology and dentin structure [44].

Our current results highlighted the strong antimicrobial effects of sodium hypochlorite alone when used with conventional needle irrigation while showing minimal additional benefits of Nd: YAG laser and PUI. Interestingly, we also demonstrated that aPDT had very little antimicrobial effects in oval canals infected with E. faecalis mature biofilms. Indeed, Nd: YAG laser, Passive Ultrasonic Irrigation (PUI), and Sodium Hypochlorite (NaOCl) achieved the highest antibacterial efficacy, producing more than 93% reduction in viable bacterial counts and near-complete elimination of the biofilm from the dentinal surface. This superior performance can be attributed to the synergistic physical and chemical effects occurring within the root canal system.

The complex morphology of oval-shaped canals presents unique challenges for traditional irrigation. Unlike round canals, the irregular geometry of oval systems creates stagnation areas and reduces wall shear stress, which facilitates debris accumulation in the buccal and lingual extensions and hinders irrigant flow to uninstrumented regions. The clinical success of disinfection is a function of both irrigant chemistry and delivery dynamics [45, 46]. While NaOCl possesses excellent tissue-dissolving and antimicrobial properties, its high surface tension often limits deep tubular penetration. Regarding the ultrasonic activation (PUI) enhances NaOCl penetration, by decreasing its surface tension, and renewal through acoustic streaming and cavitation, facilitating biofilm disruption by increasing the shear stresses and deeper diffusion of the irrigant into dentinal tubules increasing its tissue dissolution ability [1, 30, 47]. Similarly, Nd: YAG irradiation exerts combined photothermal and photomechanical effects, leading to denaturation of bacterial proteins and destruction of the biofilm matrix, while the generated heat promotes deeper penetration into the dentinal tubules [41]. The beneficial effect of Nd: YAG lies in its significant bactericidal impact in root canal disinfection which extends up to 1 mm into the dentin [10]. Notably, the finding that PUI, a widely accessible and cost-effective modality, demonstrated antimicrobial efficacy comparable to high-end laser systems, in this specific tooth model, is of significant clinical relevance. These results suggest that high-level root canal disinfection can be achieved using conventional equipment, alleviating the need for substantial capital investment in laser technology without compromising treatment outcomes.

Furthermore, the additional benefit of PUI and Nd: YAG activation can overcome the “vapor lock” effect associated with conventional needle irrigation in narrow canals. In the present study the teeth were instrumented to a size 40/04 taper, which provided sufficient volume, thus facilitating adequate fluid exchange to mitigate the vapor lock effect [48].

The marked reduction in bacterial counts achieved by the Nd: YAG laser group also corroborates previous reports. Moritz et al. [49] demonstrated a 99.16% rduction in *Enterococcus faecalis* after Nd: YAG irradiation, while Gutknecht et al. [50] achieved an average 99.92% reducion under similar parameters (15 Hz, 100 mJ). In agreement with Bergmans et al. [51] Nd: YAG laser application should not be viewed as a replacement but rather as an effective adjunct to conventional disinfection protocols, enhancing bacterial elimination without compromising safety.

Regarding PUI, the umbrella review by Suneelkumar C et al. [52], mentioned that most of the published evidence suggests that PUI achieves better antibacterial outcomes than conventional needle irrigation, though some investigations did not report any statistically significant differences between the two techniques; findings that align with the current study.

In contrast, antimicrobial photodynamic therapy (aPDT) failed to achieve significant bacterial reduction. This finding is consistent with previous reports by Hecker et al. [53], who utilized toluidine blue (635 nm for 6 min), and Souza et al. [54], who employed methylene blue and toluidine blue (660 nm for 4 min). Similarly, Muhammed et al. [55] observed limited antibacterial efficacy against E. faecalis biofilms using toluidine blue at 635 and 650 nm for 3 min. The pronounced variability in therapeutic outcomes observed across the literature is likely attributable to the lack of standardized experimental parameters, including photosensitizer (PS) chemistry, excitation wavelength, radiant exposure, pre-irradiation incubation time, and the developmental maturity of the target biofilm.

Another critical factor for the success of aPDT is the achievement of an adequate penetration depth of the PS into the complex root canal anatomy. Literature consistently reports a significant reduction in dye infiltration depth from the coronal to the apical regions, a gradient primarily dictated by morphological variations; specifically, the apical third exhibits a lower density and smaller diameter of dentinal tubules compared to the coronal segment. Furthermore, standard delivery methods, such as inoculation via a syringe and needle, often fail to achieve sufficient penetration, which may lead to suboptimal therapeutic effects [56].

The recalcitrance of *E. faecalis* to aPDT is driven by several biological resistance mechanisms. Structurally, the dense peptidoglycan layer of Gram-positive bacteria hinders the uptake of photosensitizers, while active efflux pumps further reduce intracellular dye concentrations. Within the biofilm, the extracellular polymeric substance (EPS) matrix acts as both a chemical sink and an “optical shield,” attenuating light penetration to deeper layers. Furthermore, the Type II photochemical mechanism of aPDT is oxygen dependent. The naturally hypoxic environment of mature biofilms significantly limits the production of reactive oxygen species (ROS) necessary for cell death [57]. These factors may have contributed to the inconclusive or unfavorable results highlighted in systematic reviews by Siddiqui et al. [58] and Vendramini et al. [59].

Significant bacterial reductions following antimicrobial photodynamic therapy (aPDT) have been documented in studies by Pinheiro et al. [17] and Godoy et al. [60], though their protocols feature distinct methodological parameters that may account for these favorable outcomes. Pinheiro et al. [17] utilized a model of narrow mesiobuccal roots (diameter < K-file size 15), employing a 0.01% methylene blue photosensitizer (PS) delivered via paper points. A critical aspect of their methodology was the 3-minute pre-irradiation contact time allowed before activating the 660 nm diode laser with a 600 μm fiber optic tip.

In a similar study, Godoy et al. [60] observed high aPDT efficacy when evaluating the apical 5 mm of lower premolars. Their protocol included robust chemomechanical preparation (ProTaper Next X3 and 2.5% NaOCl) prior to PS application. Notably, they utilized a longer 5-minute pre-irradiation following the intracanal injection of 0.01% methylene blue. This was followed by a 40-second irradiation period using a 660 nm diode laser and a 1 mm fiber optic tip.

Such conflicting outcomes highlight the sensitivity of aPDT performance to experimental parameters such as Pre-irradiation and irradiation time, wavelength range (typically 630–660 nm), biofilm thickness, diameter of the fiber optic tip versus the diameter of the treated root canal, whether the fiber optic tip is in contact with the canal wall or not, and the timing of aPDT if it is done before or after instrumentation [35].

The SEM observations in this study largely mirrored the CFU findings, with the notable exception of the Nd: YAG laser group. Surfaces treated with NaOCl and PUI exhibited comprehensive biofilm removal, effectively validating the quantitative CFU data. While both the NaOCl and PUI groups were categorized as Score 1, PUI demonstrated qualitatively superior debridement. Although the dentin surface may be mistaken as biofilm, these are more likely to be mineral precipitates (hydroxyapatite) or small bits of “smear layer” debris rather than a mature biological biofilm.

Interestingly, the Nd: YAG group demonstrated a pronounced reduction in CFU values, yet SEM images still revealed residual structures within the dentinal tubules. This apparent discrepancy is consistent with earlier literature. Chávez de Paz et al. [61], Gutknecht et al. [50] and Moritz et al. [49] confirmed the high bactericidal potential of Nd: YAG lasers but also reported that SEM can visualize both viable and non-viable bacterial remnants, emphasizing that structural presence does not necessarily equate to bacterial viability. Furthermore, it was noted that Nd: YAG laser irradiation may induce thermal effects resulting in dentin melting and recrystallization, producing artifacts that can resemble bacterial forms under SEM. The observed discrepancy between near-total bacterial reduction in CFU counts and the presence of residual structures in SEM images highlights the distinction between metabolic viability and morphological integrity. While the Nd: YAG laser effectively eliminated the reproductive capacity of the biofilm, the thermal energy likely induced in situ fixation of bacterial cells or protein coagulation, preserving their physical shells. Furthermore, laser-induced dentin melting and recrystallization can produce globular artifacts that resemble bacterial forms under high magnification. This underscores a primary limitation of SEM: it is a qualitative tool that cannot differentiate between viable cells, non-viable remnants, and thermal artifacts, whereas CFU remains the gold standard for functional viability assessment [62, 63].

Given that these morphological alterations and mineral crystals were not classified as active biofilm, the Nd: YAG group was assigned a Score 1.) These morphological changes are often more pronounced with longer irradiation times. However, some literature suggests that shorter cycles and less overall radiation time are sufficient for effective disinfection while minimizing thermal damage [41].

While this study found no statistically significant difference in bacterial reduction between NaOCl alone and the adjunct use of PUI or Nd: YAG laser, these results should be interpreted within the context of the study’s limitations. The ex vivo model, while controlled, may not fully replicate the anatomical complexities of a clinical setting, such as intricate isthmus areas or deep lateral canals where PUI and laser energy might provide a more distinct advantage. Furthermore, although SEM and CFU analyses are standard metrics for assessing disinfection, they may not be sensitive enough to detect subtle differences in biofilm architecture or the viability of bacteria in a ‘viable but non-culturable’ (VBNC) state. Therefore, while NaOCl remains the gold standard, the potential for incremental clinical benefits from advanced disinfection protocols cannot be entirely dismissed based on these findings alone [64].

The lack of efficacy observed in current protocols underscores the necessity for methodological refinement. It is recommended that subsequent research focuses on the development of a standardized framework for aPDT, as the absence of a consensus ‘gold standard’ to date remains a significant barrier to its clinical predictability.

It is important to acknowledge certain limitations of the present study. The study employed a simplified monomicrobial infection model using *Enterococcus faecalis*, which, while highly resistant and clinically relevant, does not fully represent the polymicrobial nature of actual endodontic infections. Future studies should therefore incorporate multispecies biofilm models and in vivo validation to better simulate clinical conditions and to assess the overall effectiveness of various disinfection methods in realistic treatment scenarios. Another limitation is the longitudinal Buccolingual sectioning of the tooth, as recommended by most of the studies, it should have been sectioned mesio-distally to inspect the uninstrumented buccal and lingual extensions in oval canals [65]. However, this did not affect our results, which also agreed with previous studies. Although laser activation significantly reduced bacterial counts in vitro, these findings do not directly translate into confirmed clinical benefits. So, additional in vivo investigations are required to determine whether laser-assisted irrigation improves clinical outcomes. A significant limitation in evaluating aPDT is the absence of a validated ‘gold standard’ protocol.

Based on these collective results, the null hypothesis was partially accepted. Significant differences were found between the control and all tested disinfection methods, except for aPDT, which did not differ significantly from the control group in its ability to remove biofilm.

## Conclusion

Within the limitations of this ex vivo mono-species study, the use of 2.5% NaOCl showed comparable antibacterial efficacy against bacterial biofilms in single rooted mandibular premolars infected with E. faecalis biofilms. Although adjunctive activation techniques such as PUI and Nd: YAG laser further enhanced bacterial reduction, their effects were not statistically superior to NaOCl alone. Additionally, aPDT using methylene blue did not show any significant antibacterial effects under the tested conditions.

Further ex vivo and in vivo studies utilizing polymicrobial models are required to validate the clinical translatability of these adjunctive disinfection protocols.

## Supplementary Information

Additional file 1: 1 Supplementary Figure and 2 graphs. The supplementary Figure shows the CFU plate for each group. The graphs demonstrate the CFU reduction.

## Data Availability

The data and materials collected in this study are available from the corresponding author when requested reasonably.

## Abbreviations

aPDT: Antimicrobial Photodynamic therapy
NaOCl: Sodium hypochlorite
E. faecalis: *Enterococcus faecalis*
CFU: Colony Forming Unit
SEM: Scanning Electron Microscope
MB: Methylene Blue
PUI: Passive Ultrasonic Irrigation
PAD: Photoactivated disinfection
WL: Working length
BHI: Brain heart infusion
TB: Toluidine blue
