# Supplementary material for: Antimicrobial activity of Nd: YAG irradiation, laser activated photodynamic therapy and passive ultrasonic irrigation on enterococcus faecalis biofilms (an ex vivo study)
Source: BMC Oral Health. 2026 May 26;26:1008. doi: 10.1186/s12903-026-08651-6 (PMC13251022; doi:10.1186/s12903-026-08651-6)
Supplement: Supplementary file 1 — Additional file 1: 1 Supplementary Figure and 2 graphs. The supplementary Figure shows the CFU plate for each group. The graphs demonstrate the CFU reduction. [file 12903_2026_8651_MOESM1_ESM.docx]

**Supplementary Figures’ legend:**

**
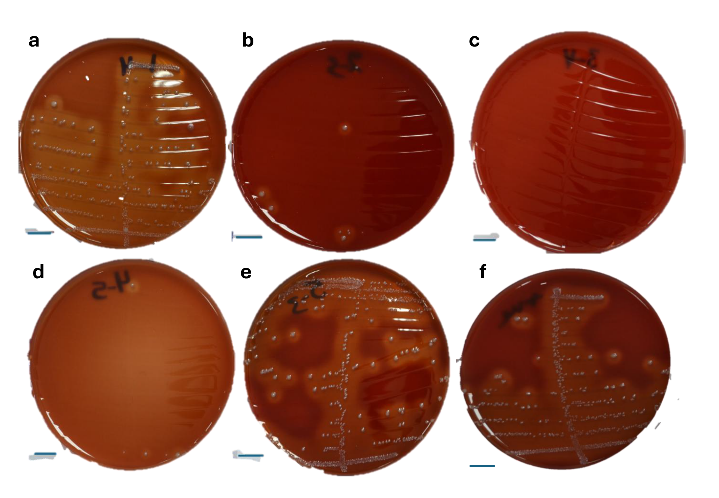
**

**Figure 1:**

Showing Enterococcus faecalis colonies on blood agar plates after the different disinfection techniques. (a) Distilled Water; (b) NaOCl; (c) PUI; (d) Nd:YAG; (e) aPDT and (f) Positive Control**.**

**
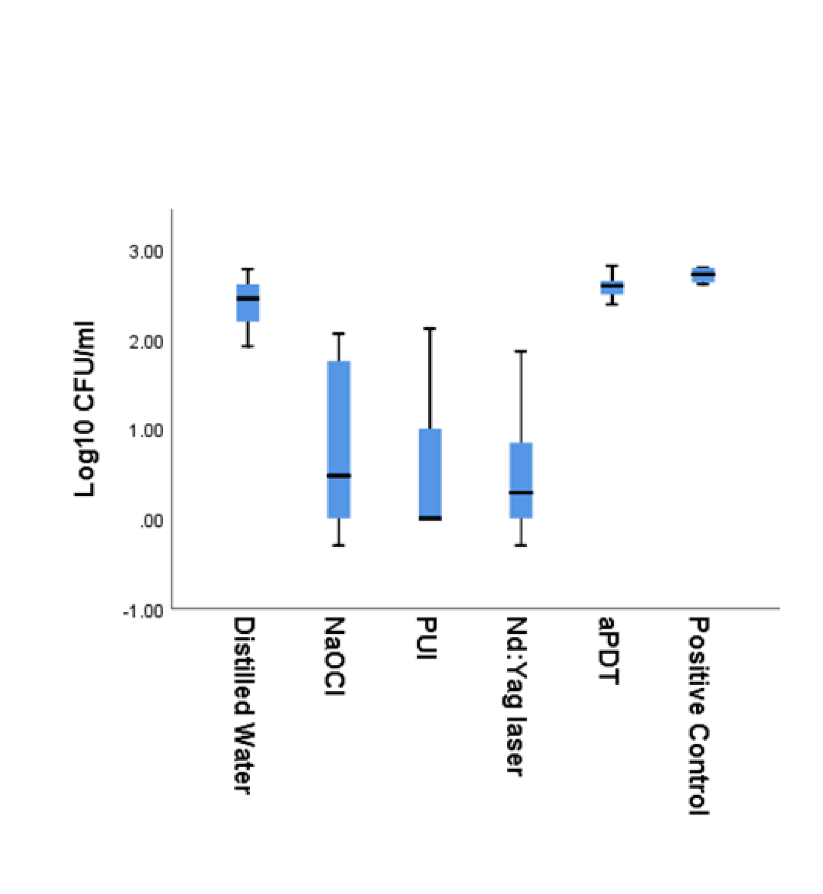
**

**Figure 2:**

E. Faecalis log_10_ CFUs in the different study groups

**
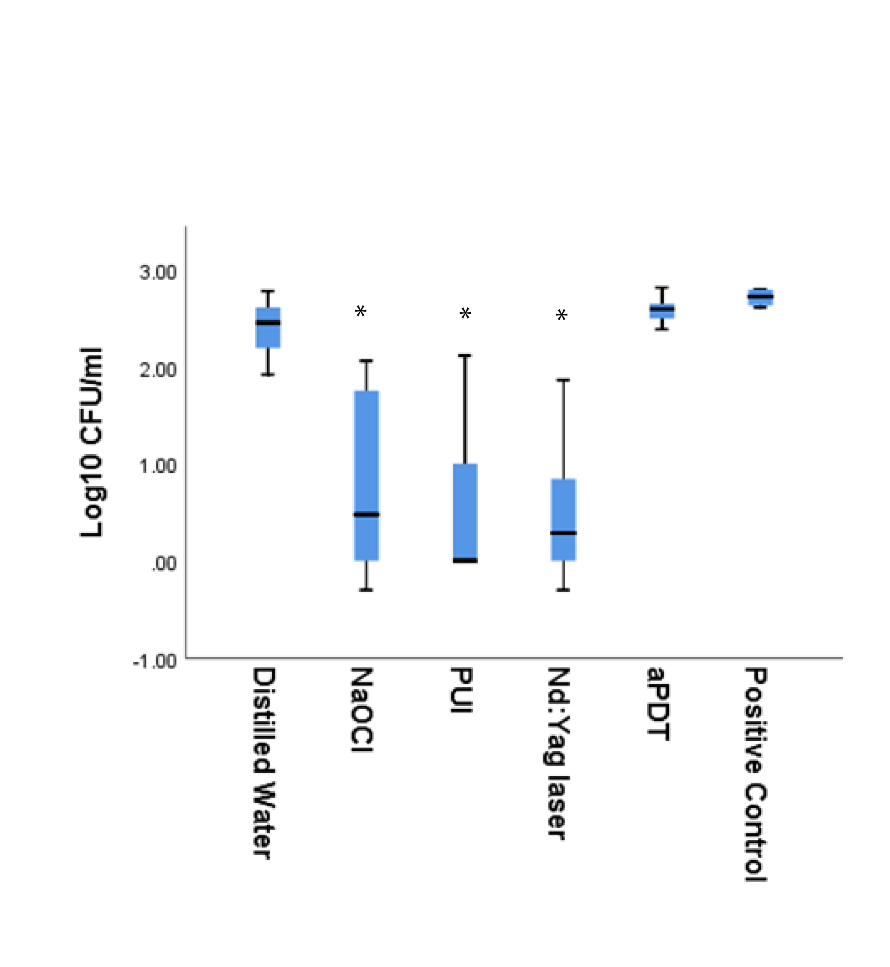
**

**Figure 3:**

Showing percent reduction (log10) of E. faecalis in comparison to counts in the positive control group after treatment with the different irrigation protocols.

* The groups demonstrated a statistically significant reduction in bacterial load compared with both the positive control and the distilled water groups (p < 0.05).
